# Nanosecond Pulsed Electric Field Suppresses Development of Eyes and Germ Cells through Blocking Synthesis of Retinoic Acid in Medaka (*Oryzias latipes*)

**DOI:** 10.1371/journal.pone.0070670

**Published:** 2013-08-06

**Authors:** Eri Shiraishi, Hamid Hosseini, Dong K. Kang, Takeshi Kitano, Hidenori Akiyama

**Affiliations:** 1 Global Center of Excellence Program on Pulsed Power Engineering, Kumamoto University, Kumamoto, Japan; 2 Bioelectrics research Center, Kumamoto University, Kumamoto, Japan; 3 Department of Pulsed Power Science, Graduate School of Science and Technology, Kumamoto University, Kumamoto, Japan; 4 Department of Biological Sciences, Graduate School of Science and Technology, Kumamoto University, Kumamoto, Japan; National Research Council, Italy

## Abstract

Application of nanosecond pulsed electric fields (nsPEFs) has attracted rising attention in various scientific fields including medical, pharmacological, and biological sciences, although its effects and molecular mechanisms leading to the effects remain poorly understood. Here, we show that a single, high-intensity (10–30 kV/cm), 60-ns PEF exposure affects gene expression and impairs development of eyes and germ cells in medaka (*Oryzias latipes*). Exposure of early blastula stage embryos to nsPEF down-regulated the expression of several transcription factors which are essential for eye development, causing abnormal eye formation. Moreover, the majority of the exposed genetic female embryos showed a fewer number of germ cells similar to that of the control (unexposed) genetic male at 9 days post-fertilization (dpf). However, all-trans retinoic acid (atRA) treatment following the exposure rescued proliferation of germ cells and resumption of normal eye development, suggesting that the phenotypes induced by nsPEF are caused by a decrease of retinoic acid levels. These results confirm that nsPEFs induce novel effects during embryogenesis in medaka.

## Introduction

Electroporation is routinely used in the field of molecular biology for the transfer of DNA to cell and tissues [1], [2]. Conventional electroporation utilizes an electric field of millisecond time order to create pores on the cell membrane to allow inflow of macromolecules [3]. High-voltage and ultra-short (nanosecond order) pulsed electric fields (nsPEFs) have recently been introduced to medicine and biology, following advances in the development of pulsed power technologies [4], [5]. Effects of nsPEFs on cells differ from millisecond or microsecond duration electric fields. Application of nsPEFs affects intracellular organelles as well as the cell membrane, offering the possibility to manipulate intracellular environment and temporarily permeabilizing the plasma membrane depending on the pulse duration [4], [6], [7]. Most studies of intracellular effects of nsPEFs have been associated with the apoptosis induction and have been performed with multiple exposures [8]. Recent discoveries have suggested new possibilities of nsPEFs use for cancer treatment [9], [10], while nsPEF application to cells and tissue has demonstrated potential to induce further unique modifications [11], [12]. This background illustrates the requirement for insight on underlying molecular mechanisms.

To elucidate possible novel effects of nsPEF, this study investigates the response of embryos of the teleost fish medaka (*Oryzias latipes*) to nsPEF. Medaka, a vertebrate of increasing use in developmental and evolutionary biology [13], [14], is a small fish with a short generation time, a transparent embryonic body, and a small genome (approximately 800 Mb −half the size of the zebrafish genome). These unique features facilitate the *in-vivo* analysis of multiple developmental processes. Transgenic techniques [15] and spotted DNA microarrays of medaka genes are available, making medaka a model fish for basic research of gene expression. The medaka has an XX/XY sex determination system like mammals, and its sex determining gene, *dmy*, has been clarified [16], [17]. The first appearance of morphological sex differentiation in medaka is a significant difference in germ cells number at stage 38 before hatching; the number of germ cells of genetic females (XX) becomes greater than that of genetic males (XY), and female germ cells subsequently enter meiosis [18], [19], [20]. Recently, we established an *olvas (Olyzias vasa)-DsRed* transgenic medaka line in which germ cells can be monitored by red fluorescent protein [21]. This transgenic medaka fish is an excellent model for the analysis of multiple biological phenomena including sex determination and differentiation.

The paired-like homeobox-containing gene, *retinal homeobox* (*rx*), plays critical roles as a transcription factor for eye development in several vertebrates [22]. In humans, mutations in *Rx* result in anophthalmia or microphthalmia. The expression pattern of *Rx* genes in different species is similar. In medaka, *rx3* mRNA is expressed in anterior neuroectoderm at late gastrula stage, while *rx2* mRNA is expressed several hours later than *rx3* in the developing optic vesicle [22], [23]. The *eyeless* mutation caused by an intronic insertion in the *rx3* homeobox gene results in the complete absence of eyes [22], [23]. However, *rx2* function has not been determined yet. The conserved *paired box containing gene 6 (pax6*) is another transcription factor which is essential for eye development [24]. In *Pax6* mutant mice, small optic vesicles were initially evaginated then degenerated during subsequent stages of development, resulting in the complete absence of eyes [25], [26]. Furthermore, retinoic acid (RA) signaling decreased in the *Pax6* mutant because, the carrying RA response element (RARE) fused to β-galactosidase (lacZ) (which identifies regions of active RA signaling), thereby inhibiting the lacZ expression in the eye by *pax6* mutation [27].

In medaka, the morpholino-based gene knockdown of *pax6* exhibited a small eye phenotype, although the appearance was dependent on concentration of the morpholino [28]. However, interactions between *rxs*, *pax6* and RA have not been determined.

RA is an active derivative of vitamin A produced by a two-step metabolic pathway in which retinol is first oxidized to retinaldehyde by alcohol dehydrogenases (ADHs), and then is subsequently oxidized to RA by *retinal dehydrogenases* (RALDHs, renamed as ALDHs) [29]. Signaling by RA is sufficient for meiosis and regulates sex-specific timing of meiosis initiation in mice [30], [31]. During embryogenesis, RA is produced by RALDH2 in the mesonephroi and then induces meiosis in embryonic ovary. Conversely, in embryonic testis, the retinoid degrading enzyme *cytochrome p450, 26 family of enzyme b1* (CYP26B1) prevents meiosis. Initiation of meiosis by RA has also been reported in chickens [32].

In this study, we exposed medaka embryos to single nsPEF and investigated its effects during embryonic development. Here, we show that nsPEF regulates gene expression and suppresses development of eyes and germ cells during embryogenesis.

## Materials and Methods

### Ethical Statement

All embryo procedures were approved by the institutional ethics committee of Kumamoto University (approval number 23–032).

### Animals

FLFII stock fish (Bioscience and Biotechnology Center, Nagoya University, Japan) were used to generate a transgenic medaka strain [33]. In this stock, genetic sex is identifiable by the appearance of leucophores at 2 dpf, which is before the onset of sex differentiation. A *sycp1 (synaptonemal complex protein 1)-GFP/olvas-DsRed* double transgenic medaka line [21, unpublished] was used to investigate the number and meiosis of germ cells. Fish embryos were maintained in ERM (17 mM NaCl, 0.4 mM KCl, 0.27 mM CaCl_2_·2H_2_O and 0.66 mM MgSO_4_, pH 7.0) at 26°C under a 14-hour light, 10-hour dark cycle. The developmental stages of the embryos were determined as described previously [34].

### nsPEF and All-trans Retinoic Acid Treatment

**Figure 1:**
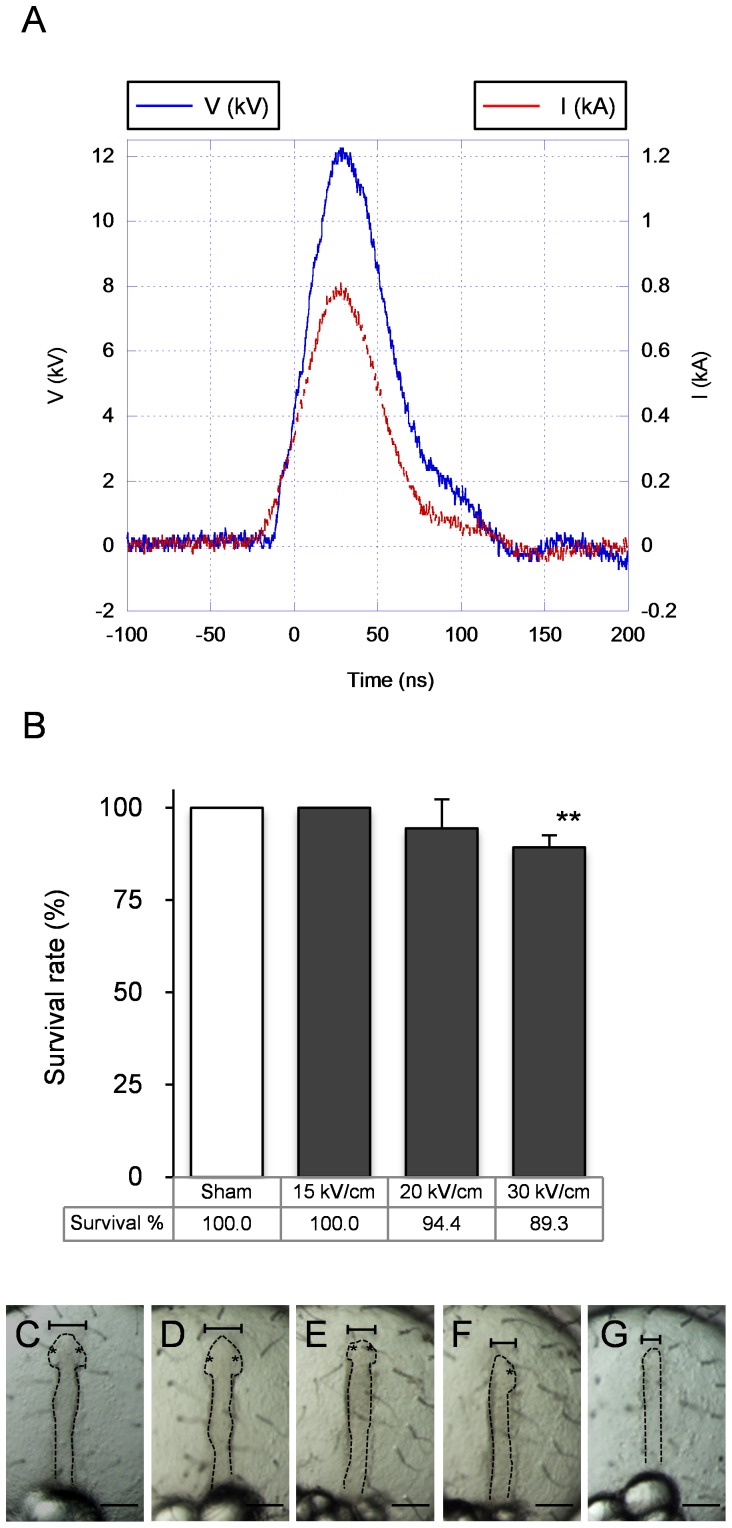
Survival rate and abnormal optic vesicle after nsPEF exposure. (A) Typical waveform with maximum voltage of 12 kV (30 kV/cm electric field in a 4-mm gap cuvette) and 60 ns pulse width at half width half maximum. (B) The survival rate after exposure. Results are expressed as the mean ± s.e.m. of average survival rate from three independent controls and exposed groups (n>29) (**P<0.01). (C-G) Abnormal optic vesicle formation after exposure. (C) Control embryo; 30 kV/cm exposed embryos with: (D) Normal optic vesicles, (E) Small optic vesicles, (F) Single optic vesicle, (G) Without any optic vesicle. Asterisks show optic vesicles. Top scales indicate width of the embryos’ head: (C) 100 µm, (D) 100 µm, (E) 75 µm, (E) 67.5 µm, (F) 50 µm. Scale bar 100 µm.

A single medaka egg at 6 hours post-fertilization (hrpf), stage 10 (early blastula) was placed with 800 µl of phosphate buffered saline in an electroporation cuvette which was equipped with two parallel aluminium electrodes with a 4-mm gap for electric field application (#5540, Molecular BioProducts). A single shot nsPEF with 60 ns full width at half maximum (FWHM) pulse duration was generated using a pulsed power modulator and applied to the embryo in the cuvette. The pulse power modulator, developed by our group and explained in references [35]–[36], consisted of a magnetic pulse compression circuit with a high speed thyristor switch system. During each experiment, voltage and current waveforms were monitored by a high voltage probe (Tektronix P6015A, USA) and a current monitor (Pearson, Model 5046, USA), respectively, and were recorded using a digital scope (Tektronix DPO4104, USA). After pulse application, each embryo was translocated in ERM solution at 26°C. The control embryos were sham exposed: they underwent all procedures except the pulse. Survival analysis was performed for the control and exposed embryo groups. At least three sets of independent experiments were performed for each group of embryos (n>29), and the average survival rate was estimated (Fig. 1B). For RA treatment, embryos after 30 kV/cm exposures were immediately reared from 6 hrpf to 9 dpf in ERM mixed with 1 nM all-trans-RA (Sigma-Aldrich, Gillingham, UK) at 26°C. Germ cells and meiotic cells were observed at 9 dpf.

### DNA Microarray

Three independent sets of experiments were carried out for the exposed and control groups. Total RNA was extracted from 20 medaka embryos at stage 18 using an RNeasy Mini kit (Qiagen, Hilden, Germany). DNA microarray hybridization and analyses following the protocol of the manufacturer, Agilent, for one-colour, microarray-based gene expression analysis (version 5.5, February 2007) were carried out with a medaka microarray tip (44 K; Agilent, Santa Clara, CA) in the Chemical Evaluation and Research Institute (Tokyo, Japan). The probe sequence was compared with NCBI’s protein database using the BLASTx program (http://blast.ncbi.nlm.nih.gov). Sequence reads with E <0.05 were assigned as putative identities. The top fifty up-regulated or down-regulated genes were annotated by biological process and cellular component based on Gene Ontology (GO) using the GO consortium (http://www.geneontology.org/) and UniProtKB/Swiss-Prot (http://www.uniprot.org/).

### Real-time PCR

Total RNA extracted from 5 pooled embryos (stage18) using ISOGEN (Nippongene, Tokyo, Japan) was reverse-transcribed by oligo (dT) priming with an RNA PCR kit (Applied Biosystems, Foster City, CA) at 42°C for 30 min [21]. Embryos were immersed in ISOGEN and placed on ice to extract total RNA. All experiments were repeated three times. The *rx2*, *pax6*, *adh5* mRNA levels were determined by quantitative real-time PCR using SYBR Green I Master (Roche Diagnostics) on a LightCycler 480 (Roche) with the following primers: forward primer for *rx2* (DDBJ accession no. AJ250405), 5′-GAGCACGGCAAGAAAAAGCA-3′; reverse primer for *rx2*, 5′-GTCATGGAGCTTCATGGTAC-3′; *pax6* (DDBJ accession no. AJ000938), 5′-CTCCATGATGCAGAACAGTC-3′, reverse primer for *pax6*, 5′-TACTCACACAGCCATTGGAC-3′; *adh5* (DDBJ accession no. AY512892), 5′-ACAGGGGTGTGTCACACAGA-3′; reverse primer for *adh5*, 5′-GTCTTGTCAGGAAGAAGGCC-3′; forward primer for *elongation factor 1 alpha* (*ef1α;* DDBJ accession no. AB013606), 5′-TGAGATGGGCAAGGGCTCCT-3′; reverse primer for *ef1α*, 5′-GCTGGGTTGTAGCCGATCTT-3′.

### Germ Cell Counting

Germ cells exhibiting DsRed fluorescence were counted for each embryo at 9 dpf under a fluorescent stereomicroscope (MZFL III; Leica Microsystems GmbH, Wetzlar, Germany). The existence of GFP-positive germ cells was assessed at the same time.

All experimental data were analyzed by Student’s *t*-test.

## Results

### Changes in the Levels of Transcripts after Single nsPEF Exposure

To investigate the effects of nsPEF on medaka embryos, we applied single nsPEF with various voltages to medaka eggs. A typical current and voltage waveform applied to the 4-mm gap cuvette containing a single egg is shown in Fig. 1A. In order to focus on influences to cell differentiation, we selected embryos of the early blastula stage (6 hrpf), which are known to have pluripotency property. Embryo survival rate was found to be voltage dependent, and even at maximum voltage, was nearly 89% (Fig. 1B). After 20 hours (at late neurula stage) from exposure of 30 kV/cm, the first morphological aberration was observed: small optic vesicles in 18% of the exposed embryos appeared (n = 34) as shown in Figs. 1 C–G. At the late neurula stage, optic vesicles could be recognized as a narrow streak after the embryonic shield became clearly visible. Although all of the controls and most of the exposed embryos showed normal bilateral optic vesicles (Figs. 1C and D), abnormal optic vesicle formation was observed among some exposed embryos such as small optic vesicles in both sides of a small head (Fig. 1E), a single optic vesicle on one side (Fig. 1F), or the absence of any optic vesicle (Fig. 1G). The head width was smaller in the exposed embryos with abnormal optic vesicles (75 µm in Fig. 1E, 67.5 µm in Fig. 1F, and 50 µm in Fig. 1G) than in control or the other exposed embryos (100 µm in Figs. 1C and D). Based on these observations, we used single nsPEF of 30 kV/cm in subsequent experiments.

**Figure 2:**
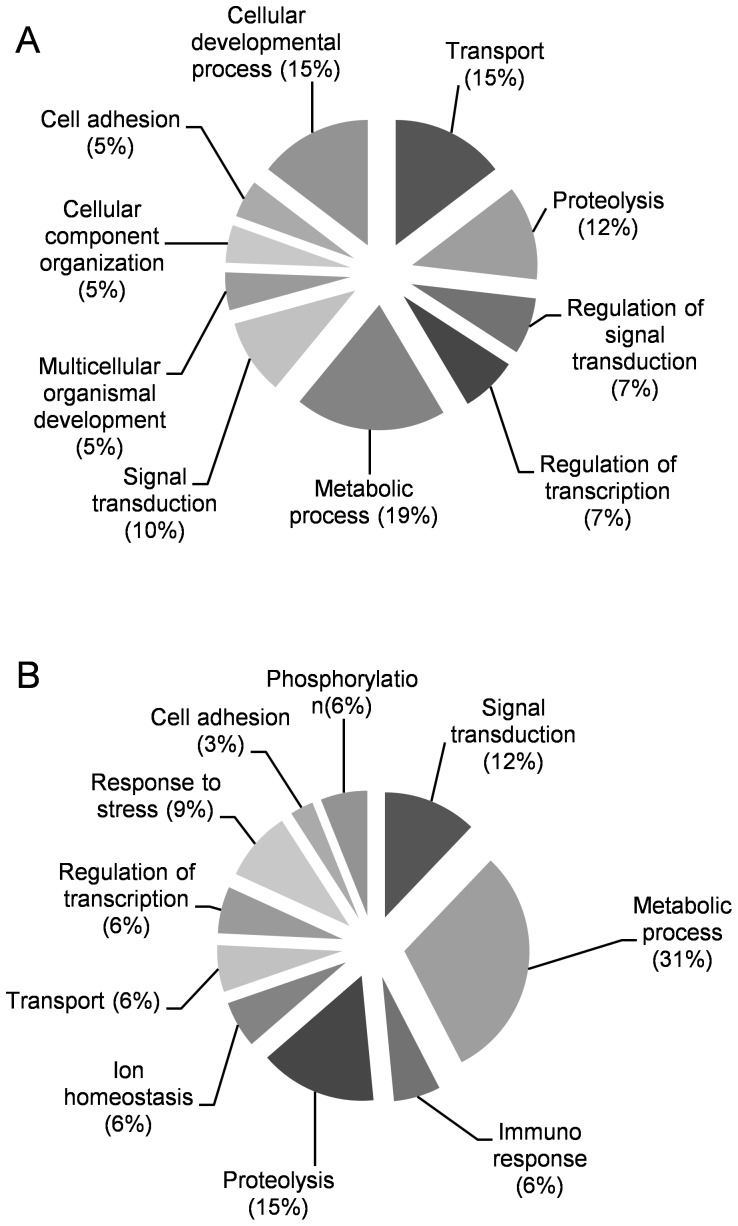
Top ten GO term distributions of top 50 genes identified by DNA microarray analysis. The down- or up-regulated genes were annotated by biological process (A, B).

Incomplete formation of optic vesicles led us to hypothesis that cell differentiation might be impaired at the transcript level by exposure to nsPEF. To investigate changes at transcript level after nsPEF application, we next performed DNA microarray analysis using the total RNAs obtained from both the exposed and control group embryos. 589 genes were identified as up-regulated by the nsPEF, while 207 genes were identified as down-regulated. They were annotated in NCBI; genes that were expressed less than half or more than 3-fold due to nsPEF application are listed in Tables S1 and S2. Gene ontology (GO) classification of the top 50 genes was also performed. The top ten GO term distributions revealed that the most specifically down- or up-regulated groups were related to the cellular developmental process or to response to stress (Figs. 2A and B, Table S3).

**Figure 3:**
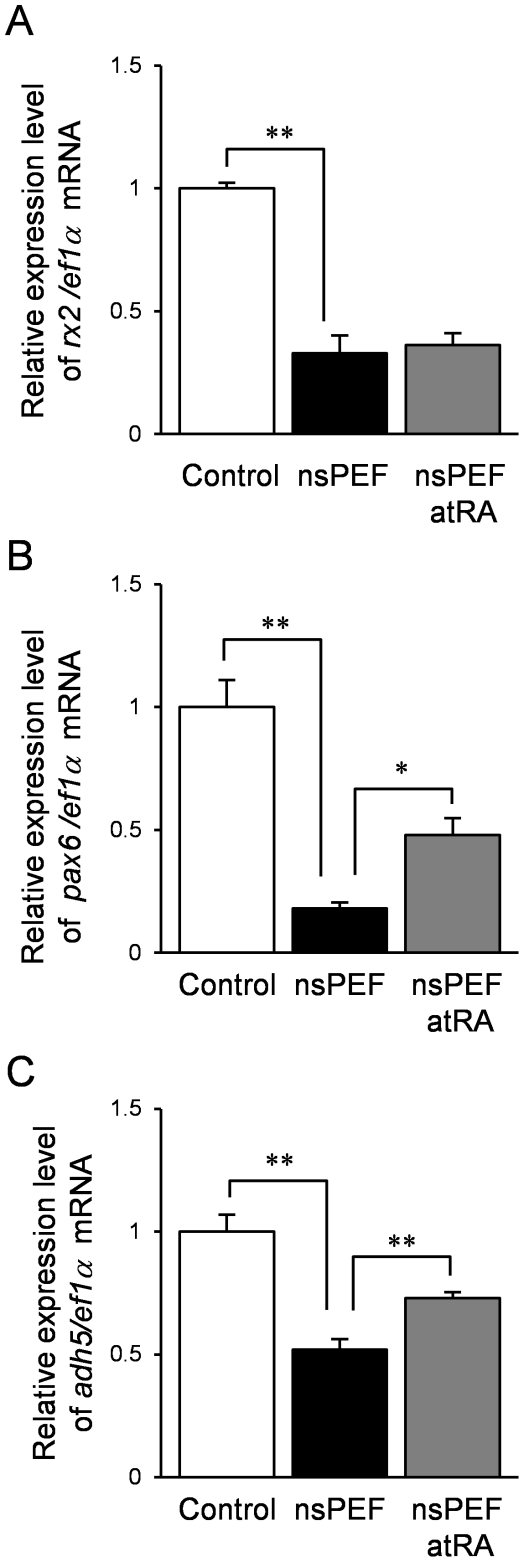
Quantitative real-time PCR of nsPEF-exposed embryos. Quantitative real-time PCR analysis of *rx2*, *pax6* and *adh5* in control, nsPEF-exposed and atRA treated eggs. Values indicate the mean ± s.e.m. of the ratio of gene expression in the exposed medaka to that of control (n = 3; *P<0.05 and **P<0.01).

Based on DNA microarray analysis, we found that expressions of *rx2* and *pax6* were down-regulated by nsPEF treatment. To confirm their expression pattern, we performed quantitative real-time PCR analysis. The mRNA levels of *rx2* and *pax6* were significantly lower in the exposed embryos compared with the control, suggesting that the single nsPEF affected eye development (Figs. 3A, B). As RA signaling in mice eye development is dependent upon *Pax6*, the results suggest the possibility that the medaka with abnormal head regions might have decreased RA signaling. The mRNA level of *adh5* also decreased in embryos of the applied group compared with the control (Fig. 3C). However, atRA treatment rescued expressions of *pax6* and *adh5* (Figs. 3B, C).

### Suppression of Germ Cell Proliferation, sycp1 Expression, and Eye Development after the nsPEF Exposure

To study the effects of single nsPEF on the development of germ cells during early gonadal differentiation period, experiments were performed using a novel *sycp1-GFP/olvas-DsRed* double-transgenic medaka line. Germ cells are highly specialized cells which form the gametes of both sexes. Meiosis is a unique division of germ cells and is achieved by assembly of synaptonemal complex proteins including central SYCP1 [37].

**Figure 4:**
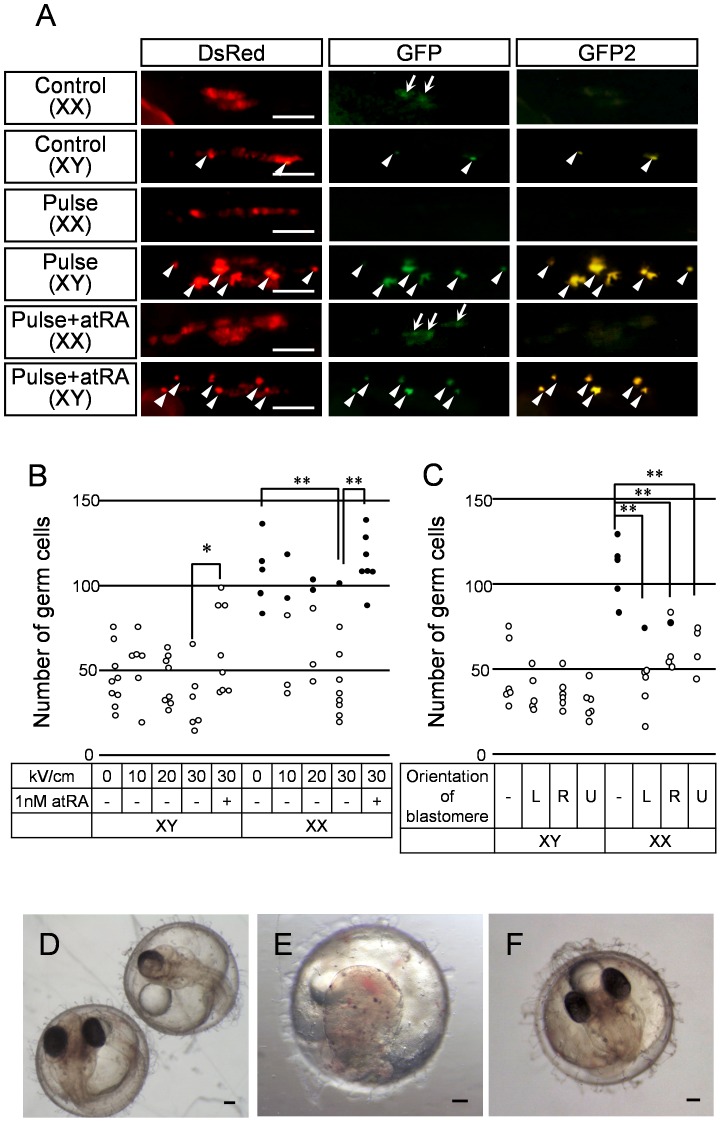
Effects of nsPEF on development of germ cells. (A) Photographs show DsRed- or GFP-positive germ cells in the individual medaka embryos at 9 dpf; control, 30 kV/cm nsPEF-exposed, and atRA-treated following 30 kV/cm exposure. Arrows and arrowheads indicate GFP-positive germ cells and autofluorescence of XY-specific leucophores that were ascertained under a different excitation wavelength (GFP2), respectively. (B) The number of DsRed-positive germ cells in control and nsPEF-exposed medaka at 9 dpf and (C) the number of DsRed-positive germ cells when the egg was placed in a different orientation between the two electrodes and exposed to 30 kV/cm nsPEF. L, R and U indicate that egg blastomeres directed towards the left electrode (the high voltage electrode), the right electrode (ground) and upward, respectively. (*n* ≥ 5; *P<0.05, **P<0.01). Black circles indicate embryos with GFP-positive germ cells and white circles refer to those without GFP-positive germ cells. (D-F) Embryos at 4 dpf; D: the left and right side photos show a control and a 30 kV/cm nsPEF exposed embryos, respectively, E: a 30 kV/cm nsPEF exposed embryo, and F: an embryo treated with atRA after 30 kV/cm exposure. Scale bar 100 µm.

At 9 dpf, the control genetic female (XX) and male (XY) medaka had different numbers of germ cells (Figs. 4A, B). GFP-positive cells were observed in the control genetic females (XX) but not in males (XY) (Figs. 4A, B). However, 9 days after exposure to nsPEF, the number of DsRed-positive germ cells tended to decrease in the genetic female (XX) medaka in a voltage-dependent manner (Fig. 4B).

Blastodermal cells existed only at a specific location (animal pole) near the yolk sphere surface at the 6 hrpf stage during nsPEF application. This asymmetry in embryonic structure might raise questions regarding result dependency on the position of the blastomere relative to the high voltage or ground electrodes; however, Fig. 4C shows that the experimental results were independent of the blastodermal cells orientation relative to the cuvette’s electrodes. This suggests that the electric fields applied to the eggs were uniform, since the electrode gap (4 mm) was considerably larger than the egg sphere (1.2 mm diameter) and each egg was carefully placed at the center of the cuvette gap.

The majority of the exposed genetic female embryos showed a fewer number of germ cells similar to that of the control (unexposed) genetic male. Also, the exposed genetic female embryos had very few GFP-positive germ cells, again similar to the control genetic male. Together, they indicate that the exposed genetic females resembled the control male (Figs. 4A, B, and C). Furthermore, we observed several dysplastic eye phenotypes at 4 dpf such as development of only a single eye (right embryo in Fig. 4D) or a completely defective eye (Fig. 4E). However, atRA treatment after nsPEF exposure entirely rescued the number of germ cells to normal, the appearance of GFP-positive cells in the genetic female (XX) medaka (Figs. 4A, B), and the resumption of normal eye development (Fig. 4F), suggesting that these nsPEF effects were due to decrease of RA levels.

## Discussion

Cellular response to nsPEF stimulus and its *in-vivo* application are of great interest in medical and biological research, while the extent of nsPEF effects is not yet well understood. Here, *in-vivo* effects of a single 60-ns PEF on embryogenesis of medaka are presented. Our results show that the nsPEF affects the development of eyes and germ cells in medaka.

The exposed embryos showed several defective eye phenotypes such as small, single, or the complete absence of eyes during early embryogenesis (Figs. 1C–G and Figs. 4D–F). *pax6* gene is known as a key regulator of vertebrate eye development [24], while in medaka *rx2* gene’s role in eye development has not yet been determined. Expression of both *rx2* and *pax6* genes significantly decreased after nsPEF exposure (Fig. 3). The eye phenotypes resulting from nsPEF exposure might be due to the suppression of *rx2-, pax6*-dependent pathway in the eye. These results also suggest that *rx2* might play some role in medaka eye development. However, atRA treatment of the exposed embryos clearly led to the resumption of normal eye development, indicating that the RA signaling system was maintained in a relatively normal state, while abnormal eye development was closely associated with decrease in endogenous RA level as well. Therefore, *rx2*, *pax6* and RA all appear related to regulate eye development. Further experiments are required to understand the inter-relationship between these three factors as multiple molecular mechanisms induced by nsPEF exposure.

RA is responsible for the sexual dimorphic initiation of meiosis in mice and chickens [31], [38], [39]. In mice, meiosis in genetic females is initiated by RA during the sex differentiation period, while this is not the case in genetic males, as RA metabolic enzyme metabolizes RA in the genetic male [40]. The sexual dimorphic timing of meiosis initiation is also similar in medaka [18]–[20]. Moreover, RA induces proliferation of primordial germ cells *in-vitro* [41]. In the present study, for the majority of the exposed female (XX) medaka, *sycp1*-positive cells vanished and germ cell number decreased, although both were rescued to normal levels by atRA treatment following exposure (Figs. 4A, B, C). Thus, we conclude that a single nsPEF suppresses germ cell proliferation by blocking synthesis of RA.

The exposed embryos were able to respond to exogenous atRA; meanwhile, nsPEF application suppressed expression of *adh5, class* III ADH (Fig. 3C and Figs. 4A–F). In mouse, oxidation of retinol to retinaldehyde is ubiquitous as it is catalyzed by ADH3 (class III ADH), which is not tissue-specific; while, is widely expressed throughout development [29]. In medaka, *adh5*, an orthologue of mammalian Class III ADH, is also ubiquitously expressed, however, whether ADH5 can induce the RA synthesis system in medaka has not yet been determined [42]. Our results suggest that nsPEF exposure affected the first step of RA synthesis, oxidation of retinol to retinaldehyde, while the exposure did not influence the signal transduction, including RA receptors. To clarify whether *adh5* suppression attributes to block RA synthesis, the roles of ADH5 on RA synthesis in medaka should be investigated.

In summary, the results clearly indicate that single nsPEF regulates development of germ cells during the sex differentiation period. Further investigation of molecular mechanisms induced by nsPEF will pave the way for future application of nsPEFs as a unique stimulus to modify embryogenesis.

## Supporting Information

Table S1 **Down-regulated genes by nsPEF exposure.**
(XLSX)

Table S2 **Up-regulated genes by nsPEF exposure.**
(XLSX)

Table S3 **Gene ontology (GO) term of top 50 genes.**
(XLSX)
